# Hormonal regulation of glycine decarboxylase and its relationship to oxidative stress

**DOI:** 10.14814/phy2.14991

**Published:** 2021-08-02

**Authors:** Ruta Jog, Guohua Chen, Jian Wang, Todd Leff

**Affiliations:** ^1^ Department of Pathology Center for Integrative Endocrine and Metabolic Research Wayne State University School of Medicine Detroit MI USA

**Keywords:** diabetes, glucagon, glutathione (GSH), glycine decarboxylase (GLDC), glycine, oxidative stress, transcriptional regulation

## Abstract

In both humans and rodent models, circulating glycine levels are significantly reduced in obesity, glucose intolerance, type II diabetes, and non‐alcoholic fatty liver disease. The glycine cleavage system and its rate‐limiting enzyme, glycine decarboxylase (GLDC), is a major determinant of plasma glycine levels. The goals of this study were to determine if the increased expression of GLDC contributes to the reduced plasma glycine levels seen in disease states, to characterize the hormonal regulation of GLDC gene expression, and to determine if altered GLDC expression has physiological effects that might affect the development of diabetes. The findings presented here show that hepatic GLDC gene expression is elevated in mouse models of obesity and diabetes, as well as by fasting. We demonstrated that GLDC gene expression is strongly regulated by the metabolic hormones glucagon and insulin, and we identified the signaling pathways involved in this regulation. Finally, we found that GLDC expression is linked to glutathione levels, with increased expression associated with elevated levels of glutathione and reduced expression associated with a suppression of glutathione and increased cellular ROS levels. These findings suggest that the hormonal regulation of GLDC contributes not only to the changes in circulating glycine levels seen in metabolic disease, but also affects glutathione production, possibly as a defense against metabolic disease‐associated oxidative stress.

## Graphical abstract

We demonstrate that the expression of glycine decarboxylase (the primary glycine degradative enzyme) is strongly induced by glucagon and that the elevation of its expression induces an increase in cellular glutathione content.

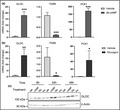

## 1 NEW AND NOTEWORTHY

Our findings suggest that the elevated expression of the primary glycine degradative enzyme, glycine decarboxylase (GLDC), contributes to the reduced levels of circulating glycine seen in diabetic and obese humans. We demonstrate that the expression of GLDC is strongly induced by glucagon and that the elevation of GLDC expression induces an increase in the cellular glutathione content.

## 2 INTRODUCTION

Among the metabolic derangements seen in obesity and type 2 diabetes are alterations in amino acid metabolism. In addition to the well‐characterized increase in circulating levels of branched‐chain amino acids in diabetic patients (Lynch & Adams, 2014), there is an equally striking reduction in the level of glycine (Adeva‐Andany et al., 2017; Yan‐Do & MacDonald, 2017). In spite of a significant amount of research, the mechanistic relationship between altered amino acid metabolism and the pathologies associated with obesity and diabetes are not fully understood.

Multiple studies have reported reduced levels of glycine in type 2 diabetes, obesity, and insulin resistance states in both animals and humans (Felig et al., 1969; Lustgarten et al., 2013; Mohorko et al., 2015; Newgard et al., 2009; Palmer et al., 2015; Takashina et al., 2016; Thalacker‐Mercer et al., 2014; Wang‐Sattler et al., 2012). In addition, interventions that improve glucose homeostasis, such as exercise (Glynn et al., 2015) and bariatric surgery (Tulipani et. al., 2016), are associated with increased glycine levels. Finally, dietary glycine supplementation has been reported to improve glucose tolerance (Gannon et al., 2002; Gonzalez‐Ortiz et al., 2001). In spite of this strong association of glycine metabolism with diabetes and related conditions, a direct role for glycine in the regulation of insulin sensitivity or glucose homeostasis has not been demonstrated.

The level of glycine in circulation is determined by a combination of nutrient influx, biosynthesis from a variety of precursors, and catabolic degradation (for a review see Wang et al., 2013). The majority of glycine degradation occurs via the glycine cleavage system, a multi‐enzyme complex located in the mitochondrial matrix that degrades glycine and produces ammonia, carbon dioxide, methylenetetrahydrofolate and NADH. The key rate‐limiting component of the glycine cleavage system is the enzyme glycine decarboxylase (GLDC), which is highly expressed in the liver, brain, kidney, and placenta.

The importance of GLDC in determining the level of circulating glycine is illustrated by inherited defects in the human GLDC gene that reduce or abolish glycine degradation and lead to elevated blood glycine levels and a variety of neurological, cardiovascular, and developmental abnormalities (Azize et al., 2014; Swanson et al., 2015). Knockout of GLDC in mice causes a significant increase in plasma glycine (Pai et al., 2015). In cultured cells, experimental induction of GLDC expression decreases glycine levels, while GLDC knockdown has the opposite effect on glycine levels (Zhang et al., 2012). These observations suggest that reduced levels of glycine seen in diabetes might be due to the elevated expression of GLDC. One of the goals of this study is to determine if GLDC gene expression is elevated in diabetes and related metabolic conditions.

The link between glycine levels and diabetes also raises the possibility that alterations in glycine metabolism, for example, reduced glycine availability, could play an active role in diabetes susceptibility or progression. This possibility is consistent with the knowledge that glycine plays a crucial role in multiple physiological processes that are related to metabolic diseases. Of particular interest in this regard is the cellular antioxidant glutathione. The last step in the glutathione biosynthetic pathway, catalyzed by glutathione synthase, is the addition of glycine to a cysteine–glutamate dipeptide. Given that the intracellular concentration of glycine is near the Km for glutathione synthase, it is possible that alterations in intracellular glycine concentrations could impact the rate of glutathione production (McCarty et al., 2018). Considering the involvement of oxidative stress in diabetes (Rani et al., 2016), a reduction in the cellular glutathione levels could alter the course of the disease. Another goal of this study is to determine if the altered expression of GLDC affects glutathione production.

We report here that GLDC is over‐expressed in animal models of diabetes, obesity, and nutritional stress, and that GLDC gene expression is strongly regulated by metabolic hormones, especially glucagon. We also observed a clear link between GLDC levels and glutathione production, although this interaction occurs by an unexpected mechanism. Our findings support the possibility that glycine catabolism plays a functional role in the development and progression of metabolic disease.

## 3 MATERIALS AND METHODS

### 3.1 Animal experiments

All animal experiments were reviewed and approved by the Wayne State University IACUC committee and carried out under the institutional guidelines for ethical animal use.

The leptin receptor‐deficient db/db and control db/+ mouse livers were a gift from Dr. Jeimei Wang, Department of Pharmaceutical Sciences, Eugene Applebaum College of Pharmacy and Health Sciences, Wayne State University, Detroit, MI. The livers were dissected from 9‐week‐old, male mice fed standard mouse chow after weaning. The db/db and db/+ mice were age‐ and gender‐matched littermates.

For the diet‐induced obesity experiments, male C57BL6/J mice were either fed a diet containing 45% calories from fat (Research Diets D12451) or 10% calories from fat (Research Diets D12450K) for 16 weeks. At the end of the study duration, livers were harvested and snap‐frozen. For the fasting experiments, 12‐week old male C57BL6/J mice on a chow diet were either fed ad lib or fasted for 12 h. All mice were sacrificed at the same time of the day between 8:00 and 10:00 AM. All animals had unrestricted access to water.

#### 3.1.1 mRNA isolation and quantification

Liver tissue (50–100 mg) was lysed and homogenized in 1 ml of TRIzol (Invitrogen) using a TissueRuptor II device (Qiagen). The samples were centrifuged at 12000 × g for 5 min at 4℃ to remove non‐homogenized tissue and fat. RNA isolation using TRIzol was carried out according to the manufacturer's protocol. For RNA isolation from cultured cells, total RNA was isolated after treatment with hormones using the PureLink RNA kit (Invitrogen). The total RNA isolated was quantified by absorbance at 260 nM and only samples with total RNA A260/230 ratio of above 1.8 were used for cDNA synthesis.

cDNA synthesis from 1.0 to 1.5 µg of total RNA was carried out in accordance with the manufacturer's instructions (Applied Biosystems). PCR primers used to detect mRNA abundance were purchased from Integrated DNA Technologies (IDT, Coralville, IA). The sequences of the primer sets are available upon request. Primers were designed to span exon/exon boundaries to reduce the signal from potential genomic DNA contamination. cDNA levels were determined using low ROX ABsolute QPCR mix with SYBR green (Thermo Fisher). Appropriate species‐specific PPIB or TBP were used as reference genes. Assays were performed in experimental duplicates on Stratagene Mx 3000P and the relative mRNA fold changes were quantified using the comparative cycle threshold 2^−∆∆Ct^ using either the PPIB or TBP genes as reference genes.

For cultured cells, primary rat hepatocytes (1.6 million per well) were plated immediately after isolation onto collagen‐coated 6‐well plates and cultured as described above. Forty‐eight hours after plating, hepatocytes were treated with either dibutyryl cAMP, glucagon, and/or insulin at concentrations and time durations indicated in the figure legends. At the end of the treatment period, cells were harvested and RNA extraction, cDNA synthesis and qRT‐PCR were performed as above.

#### 3.1.2 Western blotting and antibodies

For liver, 50–100 mg of tissue was homogenized in 500 µl of RIPA lysis buffer (Thermo Scientific) containing protease inhibitor cocktail. After homogenization, the tissue was centrifuged at 14000 X g for 20 min at 4℃. The cleared supernatant was carefully transferred to a new microcentrifuge tube.

For cultured cells, after washing twice with ice‐cold PBS, cells were collected in PBS using a cell lifter and centrifuged at 10000 rpm for 10 s at 4℃. Protein concentrations were determined with bicinchoninic (BCA) protein assay kit according to the manufacturer's instruction (Pierce Biotechnology). Forty micrograms of protein were mixed with loading dye, heated at 99℃ for 5 min, and run on a 10% SDS‐PAGE gel and transferred to a nitrocellulose membrane. The membrane was incubated in 5% non‐fat milk in 1X PBS‐T buffer or 5% BSA in 1X TBS‐T (when working with phospho‐CREB antibody) at room temperature for 2 h to block non‐specific binding and then probed with specific primary antibodies at 4℃ overnight. Membranes were washed three times for 10 min each with 1X PBST or 1X TBS‐T to remove unbound antibody and then incubated with HRP‐conjugated secondary antibody at room temperature for 1–2 h. Following this incubation, blots were washed three times for 10 min each with 1X PBST or 1X TBS‐T to remove the unbound secondary antibody. Protein bands were visualized with enhanced chemiluminescence reagents (Perkin Elmer). Primary antibodies were: GLDC (Sigma‐Aldrich, HPA002318), phospho‐CREB (Cell Signaling Technology, #9191S), total CREB (Cell Signaling Technology, #4820S), GAPDH (Santa Cruz) and Actin (Sigma‐Aldrich), and β‐tubulin (Cell Signaling Technology, #2146). Primary antibodies were used at a 1:1000 dilution and secondary antibodies at a 1:5000 dilution.

#### 3.1.3 Cell culture and establishment of stable cell lines

*Established Cell Lines* ‐ H4IIE (ATCC), Huh7 (a kind gift from Dr. Wanqing Liu, Department of Pharmaceutical Sciences, Wayne State University, Detroit, MI), HEK293A (Invitrogen), and HEK293T (ATCC) cells were maintained in DMEM supplemented with 100 U/ml of penicillin and 100 µg/ml of streptomycin (all purchased from Life Technologies) and 10% fetal bovine serum (Denville Scientific). Cells were incubated in a humidified 5% CO_2_ atmosphere at 37℃. Multiple cell lines were used to satisfy various experimental and technical requirements, such as the expression level of GLDC and transfectability.

*Primary rat hepatocytes* ‐ Adult male Sprague Dawley rats (180–200 gm) were used for the isolation of primary hepatocytes. Hepatocytes were isolated using a two‐step collagen perfusion method as described (Kocarek & Reddy, 1996). Immediately after isolation, hepatocytes were plated onto type I bovine collagen (Advanced BioMatrix) coated plates and cultured in Williams’ E medium (Gibco, A12176‐01) supplemented with 10% FBS, 100 nM Novolin (Novo Nordisk), 0.1 µM triamcinolone acetonide, 0.02 M glutamine, 100 U/ml of penicillin, and 100 µg/ml of streptomycin. Six hours after plating, the hepatocytes were overlaid with Matrigel (Corning) diluted 1:50 in maintenance media (Williams’ E supplemented as above, but without FBS). Additional maintenance media (devoid of Matrigel) was added the following day. Forty‐eight hours after isolation, media was changed to Williams’ E supplemented with 0.02 M glutamine, 100 U/ml of penicillin, and 100 µg/ml of streptomycin, and treated as described.

*Construction of stable cell lines* ‐ For stable shRNA‐mediated knockdown of GLDC, the pLKO.puro lentiviral small‐hairpin RNA vectors against human GLDC (TRCN0000303371) were obtained from Sigma‐Aldrich. The recombinant lentivirus was produced as described (Chen et al., 2018). For the stable knockdown of GLDC, HepG2 cells were infected with pLKO.puro‐GLDC and selected with 2 µg/ml of puromycin. Puromycin‐resistant cells were validated for altered GLDC expression by western blotting. For CRISPR mediated mutation of GLDC, the sgRNA sequence was determined as described (Lucas et al., 2018). The designed sgRNA sequence GGGTCTTTTCAAACGGATGT was annealed and cloned into pSpCas9(BB)‐2A‐Puro (Addgene) and used for the generation of the targeting vector which was transfected into HEK293A cells using lipofectamine 2000 (Invitrogen). The puromycin‐resistant cell clones were selected in 1 µg/ml of puromycin and screened for the loss of GLDC protein expression by western blotting.

*Transient transfections and reporter assays* ‐ The construction of the luciferase reporters for GLDC promoter is described elsewhere (Chen et al., 2018). For Huh7 cells, approximately 1 × 10^5^ cells in 0.5 ml of supplemented DMEM were plated per well in a 24‐well plate. Eighteen hours after plating, the cells were transfected with 1.5 µl of Lipofectamine 2000 (Thermo‐Fisher) and 75 ng of firefly luciferase reporter plasmid, 75 ng of pBlueScript, 50 ng EGFP‐N1 (Addgene), 300 ng pcDNA3.1‐Srebp1c expression plasmid (Addgene), and 0.5 ng per well of the Renilla luciferase control plasmid pRL‐CMV (Promega). Six hours later, fresh media was added to the cells. Forty‐two hours after transfection, media was changed to serum‐free DMEM for 6 h, then cells were lysed and both firefly and Renilla luciferase activities were measured using Dual‐luciferase reporter assay system (Promega) on a CLARIOstar plate reader (BMG LabTech). For each sample, the firefly luciferase value was normalized to the corresponding Renilla luciferase value. For the transient transfection of HEK293T, 800 ng pcDNA3.1‐Srebp1c, 100 ng pcDNA3.1, and 100 ng EGFP‐N1 were transfected using Lipofectamine 2000. Media changes and cell harvesting were performed as described above. Changes in GLDC endogenous mRNA were measured by qRT‐PCR.

*siRNA*‐*knockdown experiments* ‐ Primary cultures of rat hepatocytes were transiently transfected with siRNA against GLDC, CREB1, and ATF1 (Dharmacon). Hepatocytes were plated on collagen‐coated 12‐well plates (6 × 10^5^ cells per well). Cells were transfected 6 h after plating using METAFECTENE PRO (Biontex Laboratories GmbH, Munich, Germany) according to the manufacturer's instructions. After overnight transfection, the culture media was replaced with fresh maintenance media containing Matrigel (1:50 dilution) and the cells were incubated for an additional 48 h (with one media change) and then treated with dibutyryl cAMP (Sigma‐Aldrich, D0627) for 12 h before harvesting.

The results presented for all cell culture‐based experiments are derived from three independent experiments.

#### 3.1.4 Glutathione measurements

Total glutathione was measured using an enzymatic recycling method as described (Irfan Rahman AKSKB, 2006). Briefly, HepG2 and HEK293A cells were cultured for 48h in GMEM media (Gibco) supplemented with 10% FBS, 0.4 M serine and 0.4 M glycine, 2 mM glutamine, penicillin, and streptomycin. Cells were washed extensively with ice‐cold PBS, lysed by freeze‐thaw cycles, and the lysates cleared by centrifugation. After the conversion of the glutathione in the lysate to the reduced form (GSH), total GSH content was measured using 5,5'‐dithiobis‐(2‐nitrobenzoic acid) DTNB. DTNB is reduced to 2‐nitro‐5‐thiobenzoate which was quantified by absorbance at 412 nm in CLARIOstar (BMG LabTech) 96‐well plate reader. Total GSH was normalized to the amount of protein in a separate set of samples plated, processed, and harvested at the same time.

In some experiments, total glutathione was measured using the GSH/GSSG‐Glo kit (Promega). Cells were plated at 10000 cells/well in a 96‐well white wall, clear‐bottom plate, and cultured for 48h in GMEM media supplemented with 10% FBS, 0.4 M serine, 0.4 M glycine, 2 mM glutamine, penicillin, and streptomycin. After washing in ice‐cold, calcium/magnesium‐free PBS, total glutathione levels were measured using the instructions provided by the manufacturer. The results presented for glutathione measurements are representative of three independent experiments.

#### 3.1.5 Reactive Oxygen Species (ROS) measurement

The cellular ROS content was measured using a fluorescence‐based assay using the cell‐permeable CM‐H_2_DCFDA dye (Invitrogen) according to the manufacturer's instructions. Briefly, cells were plated in black wall, clear bottom 96‐well plates at 15,000 cells/well in DMEM complete media, and cultured for 24 h. An oxidative stress inducer, tert‐butyl hydrogen peroxide (tBHP) (Sigma‐Aldrich) was used in some experiments to increase oxidative stress, as indicated. For the assay, cells were transferred to FluoroBrite media (Invitrogen) and exposed to 10 µM CM‐H_2_DCFDA for 30 min at 37℃. After washing with PBS, fluorescence was measured using a CLARIOstar plate reader at Ex/Em of 492–495/517–527 nm. The fluorescence intensity was normalized to the total protein from the same wells measured by the Bradford method. The results presented for ROS measurements are representative of three independent experiments.

#### 3.1.6 Statistical analysis

Data are presented as means with error bars representing standard deviation. Statistical significance was evaluated using the two‐tailed Student's t‐test or ANOVA. All statistical analyses were performed using GraphPad Prism software.

## 4 RESULTS

### 4.1 GLDC expression is altered by metabolic stress

The reduced levels of circulating glycine observed in diabetic and obese individuals suggest that either the glycine biosynthetic or glycine degradative pathways are altered by metabolic stress. A primary determinant of the level of circulating glycine is the hepatic glycine cleavage system and its rate‐limiting enzyme glycine decarboxylase (GLDC). To explore the possibility that GLDC expression is a determinant of altered glycine levels in diabetes and other metabolic stress states, we measured hepatic GLDC expression in several mouse models: db/db mice, high fat diet‐induced obese mice, and normal mice after a prolonged fast.

**FIGURE 1:**
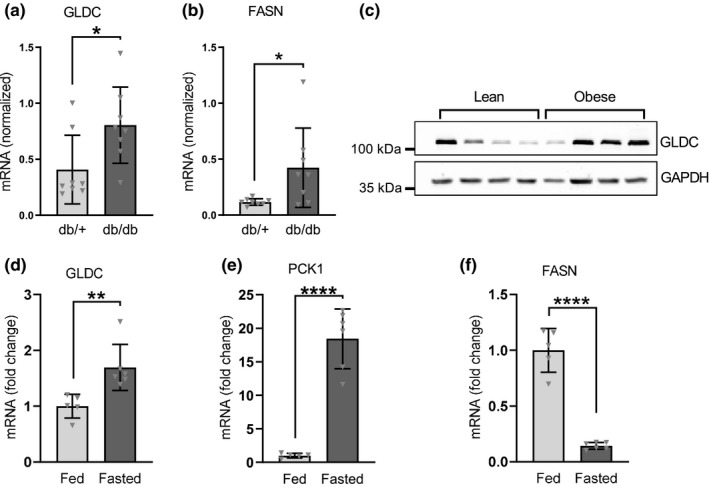
GLDC expression is elevated in mouse models of diabetes, obesity, and nutritional stress. (a) Hepatic GLDC and (b) hepatic FASN mRNA levels from 9‐week‐old db/+ and db/db male mice. Transcript abundance was quantitated by qRT‐PCR using TBP as a reference gene. Data are means ±standard Deviation, n = 8; * = *p*<0.05. (c) Western blot of GLDC protein from livers of lean and diet‐induced obese mice. (D, E, F) C57BL6/J mice were fasted for 12 hours and hepatic mRNA abundance was measured by qRT‐PCR for GLDC, PCK1, and FASN. PPIA was used as a reference gene. Data are means ±standard deviation, n = 5–6; **p < 0.01, ****p < 0.0001.

The db mouse is a standard genetic model of diabetes that exhibits insulin resistance, hyperinsulinemia, and hyperglucagonemia (Burke et al., 2017). When 9‐week old male db/db mice were compared with db/+ littermate controls, we found that hepatic GLDC mRNA levels were significantly elevated (2.0‐fold, *p *= 0.028) in the diabetic group (Figure 1A). For comparison, we also measured fatty acid synthase (FASN) mRNA which was elevated in db/db mice as expected (Figure 1B) (Assimacopoulos‐Jeannet et al., 1995; Eissing et al., 2013; Kirchner et al., 2016; Shillabeer et al., 1992).

To determine whether GLDC expression was similarly elevated in diet‐induced obesity, we examined hepatic GLDC protein levels in C57BL6/J mice fed either a low‐fat (10% calories from fat) or a high‐fat (45% calories from fat) diet for 16 weeks. Hepatic GLDC protein levels were moderately elevated (1.8‐fold elevation) in the livers of the high‐fat‐fed mice compared to controls (Figure 1C), although this difference was not statistically significant. These findings are consistent with data from the GEO profiles public database (accession number: GSE52333), where an mRNA profiling experiment comparing hepatic gene expression patterns in mice maintained on high‐fat or low‐fat diets, displayed a significant elevation (*p *= 0.005) in GLDC transcript abundance in obese versus lean mice (Eckel‐Mahan et al., 2013; Murakami et al., 2016). Together these findings indicate that GLDC expression is elevated in the states of metabolic stress such as diabetes and obesity.

To determine if the nutritional stresses of fasting also affect GLDC expression, we examined the effect of a 12 h fast on hepatic GLDC gene expression. As shown in Figure 1D, hepatic GLDC mRNA was significantly elevated (*p *= 0.0081) after 12 hours of fasting. As controls to indicate metabolic status, we also measured mRNA levels of the gluconeogenic gene PEPCK (phosphoenolpyruvate carboxykinase, PCK1) and the lipogenic gene fatty acid synthase (FASN), which as expected were stimulated and repressed, respectively, by fasting (Figure 1E and F) (Chi et al., 2020; Schupp et al., 2013). These results suggest that GLDC gene transcription is regulated by either the nutrient levels or the hormonal changes associated with fasting.

### 4.2 Hormonal regulation of GLDC expression

The findings presented above clearly show that hepatic GLDC gene expression is altered in a variety of physiologic states including nutritional stress (fasting) and metabolic stress (diabetes and obesity). These findings raise the possibility that GLDC gene expression is under the direct regulatory control of specific hormonal or nutritional signals. To explore this possibility, we conducted a survey of likely hormones and nutrients for their ability to regulate GLDC gene transcription. The following section describes the findings from the two primary candidate hormones, glucagon, and insulin.

**FIGURE 2:**
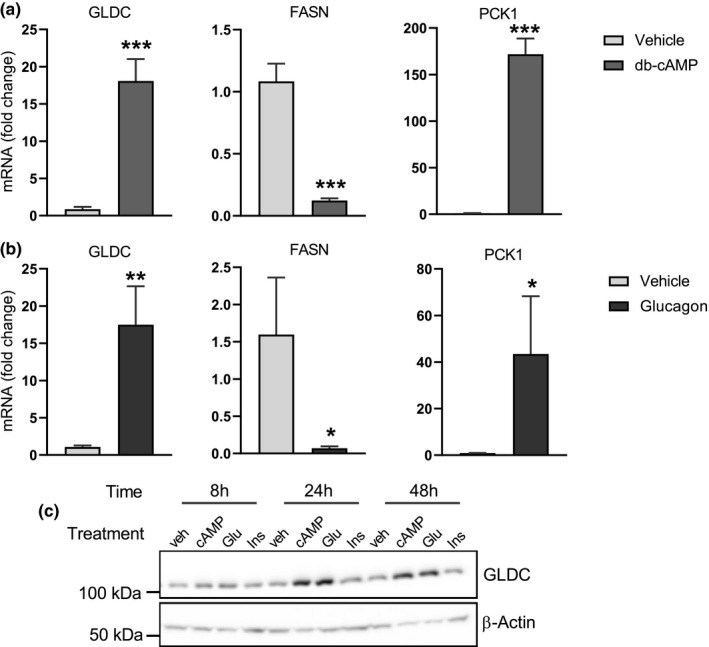
GLDC expression is stimulated by glucagon and cAMP. (a, b) Primary rat hepatocytes were treated with (a) either vehicle or db‐cAMP (100 µM) and (b) either vehicle or glucagon (100 nM) for 12h, and GLDC, FASN, and PCK1 mRNA abundance was analyzed by qRT‐PCR. PPIB was used as a reference gene. Data are the mean (n = 3) ± standard deviation; *p < 0.05, **p < 0.01, ***p < 0.001. (c) Primary rat hepatocytes were treated with either vehicle, db‐cAMP (100 μM), glucagon (100 nM) or insulin (100 nM) for 8, 24, and 48 h and GLDC protein was analyzed by western blot.

The fact that GLDC levels are elevated by fasting suggests that glucagon might be a key regulator of GLDC gene expression. This possibility was examined by treating primary rat hepatocytes with glucagon and measuring GLDC mRNA and protein levels. As shown in Figure 2A and 2B, both cAMP (the second messenger for glucagon signaling) and glucagon itself, had strong stimulatory effects on GLDC mRNA levels – 18‐fold, (*p *= 0.0005) and 17‐fold (*p *= 0.0053), respectively. GLDC protein amounts were also increased by both glucagon and cAMP (Figure 2C).

**FIGURE 3:**
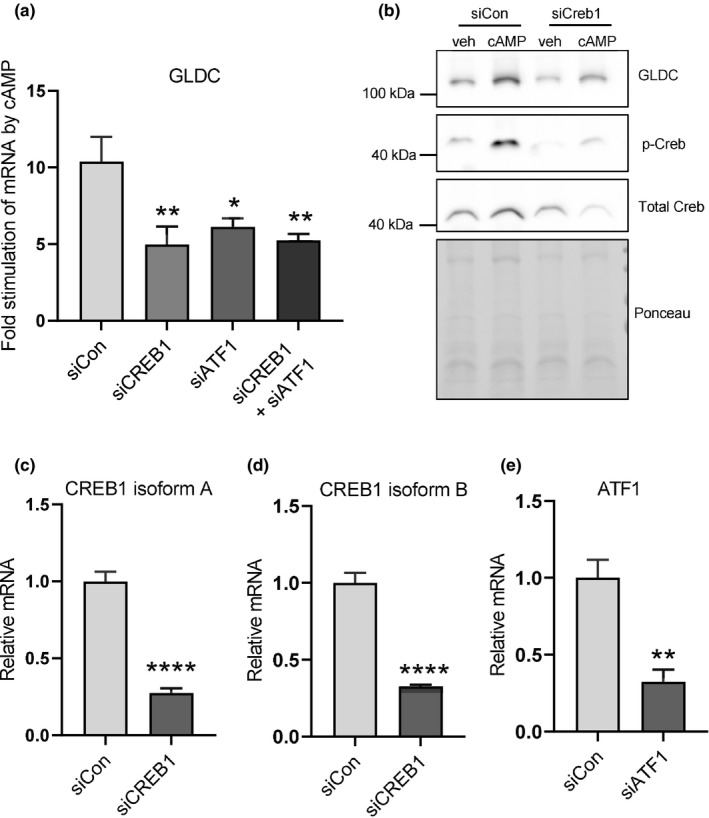
CREB1 and ATF1 mediate cAMP‐dependent activation of GLDC. (a) GLDC mRNA abundance after cAMP treatment in primary rat hepatocytes transfected with siRNA against either CREB1 or ATF1 or both as indicated. (b) Western blot showing GLDC, CREB1, Phospho‐CREB protein levels in primary rat hepatocytes transfected with the indicated siRNAs for 48h and then treated with either vehicle (veh) or db‐cAMP (100 µM) for 20h. (c, d, & e) mRNA levels of the indicated CREB isoforms showing knockdown efficiency of the siRNAs used in panels A & B. Data are means (n = 3) ± standard deviation; *p < 0.05, **p < 0.01, ****p < 0.0001.

Glucagon induces transcriptional changes in many hepatic genes via cAMP‐dependent PKA phosphorylation of the transcription factor CREB. To determine if the stimulation of GLDC expression by cAMP seen in Figure 2A was mediated by CREB, we conducted the CREB knockdown experiments shown in Figure 3. RNAi‐mediated knockdown of CREB isoforms and ATF1 reduced the ability of cAMP to stimulate GLDC gene expression, as seen at both the mRNA (Figure 3A) and protein level (Figure 3B). The observation that the double knockdown of CREB and ATF1 did not produce a stronger reduction than the knockdown of either gene individually (Figure 3A) suggests that the relevant transcription factor configuration regulating GLDC is a CREB/ATF1 heterodimer. This would be similar to the regulation of other metabolic genes by PKA‐mediated signaling, such as PEPCK and glucose‐6‐phosphatase (Titchenell et al., 2017).

Glucagon is clearly a potent regulator of GLDC transcription and is likely to be the primary mediator of the induction of GLDC expression during a fast. It may also mediate the elevated expression seen in db/db mice, which are known to have high circulating levels of glucagon (Burke et al., 2017). However, we also observed higher levels of GLDC in high‐fat fed‐mice which are insulin resistant and likely to have elevated insulin levels, suggesting that at least under some circumstances, insulin might also regulate GLDC expression.

**FIGURE 4:**
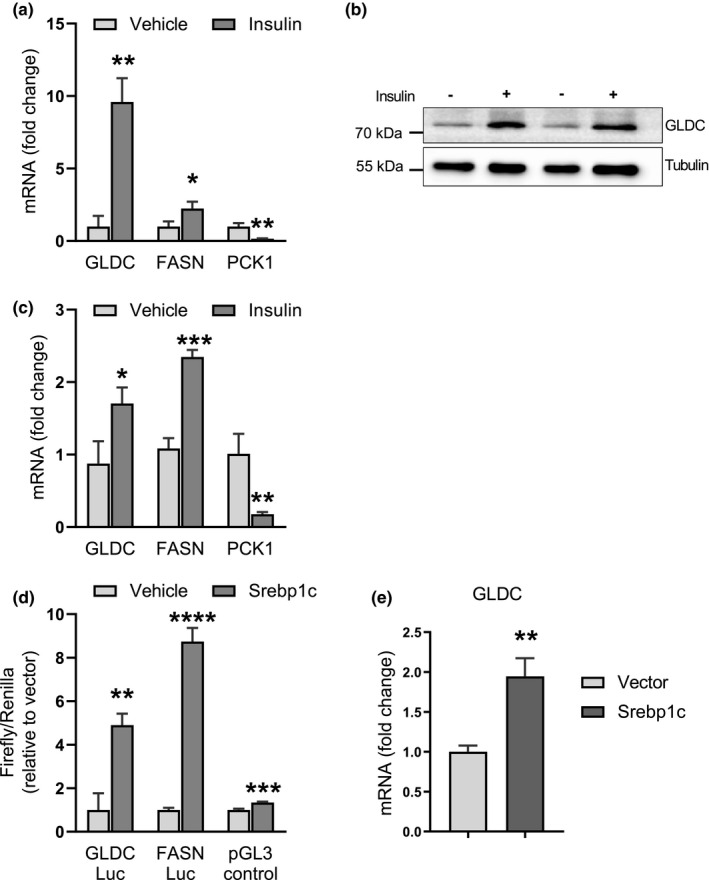
GLDC expression is stimulated by insulin and SREBP‐1c. (a) mRNA level of GLDC in H4IIE rat hepatoma cells treated with insulin (100 nM) for 8 h. FASN and PEPCK (PCK1) mRNA levels were measured as controls for hormone responsiveness. Data are means (n = 3) ± standard deviation; *p < 0.05, **p < 0.01. (b) Western blot of GLDC protein levels after a 24h treatment with insulin (100 nM). (c) GLDC, FASN, and PEPCK mRNA levels in primary rat hepatocytes treated with insulin (100 nM) for 12h. Data are means (n = 3) ± standard deviation; *p < 0.05, **p < 0.01, ***p < 0.001. (d) GLDC transcriptional activity in Huh7 human hepatoma cells transfected with GLDC promoter‐luciferase reporter plasmid and a SREBP1c expression vector as indicated. FASN and pGL3 control luciferase reporters were transfected as controls. Data are means (n = 3) ± standard deviation; **p < 0.01, ***p < 0.001, ****p < 0.0001. (e) Endogenous GLDC mRNA levels in HEK293T cells transfected with an SREBP1c expression vector. Data are means (n = 3) ± standard deviation; **p < 0.01.

To examine this possibility, we measured GLDC gene expression in H4IIe rat hepatoma cells after treatment with insulin. In these cells, we observed a striking elevation of GLDC transcript levels after an 8‐h treatment (9.6‐fold, *p *= 0.0012) (Figure 4A) and an increase in protein abundance (Figure 4B). These results demonstrate that GLDC expression can also be stimulated by insulin. A similar insulin‐mediated stimulation (*p *= 0.019) of GLDC transcription was observed in primary hepatocytes (Figure 4C). As controls to indicate functional hormone responsiveness of the cells, we also measured mRNA levels of the gluconeogenic gene PEPCK (phosphoenolpyruvate carboxykinase, PCK1) and the lipogenic gene fatty acid synthase (FASN).

SREBP1c is known to mediate insulin action on gene expression in the liver (Wang et al., 2015). We found that the GLDC promoter contained numerous SREBP1 binding sites using “MatInspector” feature of the Genomatix software. The software identified both E‐box and SRE putative SREBP1 binding sites in the human, mouse, and rat GLDC promoters. SREBP1c can bind to both E‐box and SRE motifs (Kim et al., 1995). In addition, we extracted SREBP1c ChIP‐Seq data from the ENCODE/SYDH database for insulin‐treated HepG2 cells (Reed et al., 2008) and found an SREBP1c binding signal 600 nucleotides upstream of the GLDC transcriptional start site.

To confirm that SREBP1c is a functional regulator of GLDC gene transcription, we examined its effects on a GLDC promoter‐luciferase reporter assay in transiently transfected Huh7 cells. As shown in Figure 4D, the introduction of SREBP1c stimulated GLDC transcription by 4.9‐fold (*p *= 0.0019). Fatty acid synthase (FASN) is known to be regulated by SREBP1c‐ and was included as a positive control. Additionally, we examined the effect of ectopic expression of SREBP1c on the expression of the endogenous GLDC gene in HEK293T cells (Figure 4E). We observed a significant SREBP1c‐dependent increase in GLDC mRNA levels (2‐fold, *p *= 0.0024). Together, these data demonstrate that SREBP1c regulates GLDC transcription and suggest that the stimulation of GLDC transcription by insulin is mediated by an SREBP1c‐dependent mechanism.

### 4.3 GLDC expression is linked to glutathione production and redox status

Glycine is a component of the antioxidant glutathione, a tripeptide composed of cysteine, glutamate, and glycine. The addition of glycine to the cysteine‐glutamate di‐peptide by the enzyme glutathione synthase is the final step in its synthesis. Given the key role of GLDC in regulating glycine availability, we hypothesized that elevated GLDC enzyme levels would lead to a reduction in glycine availability and consequent reduction in the rate of glutathione synthesis. Likewise, a reduction in GLDC activity would cause an increase in glycine availability and stimulate glutathione synthesis. To test this hypothesis, we measured total glutathione levels in cells with reduced GLDC enzyme levels.

**FIGURE 5:**
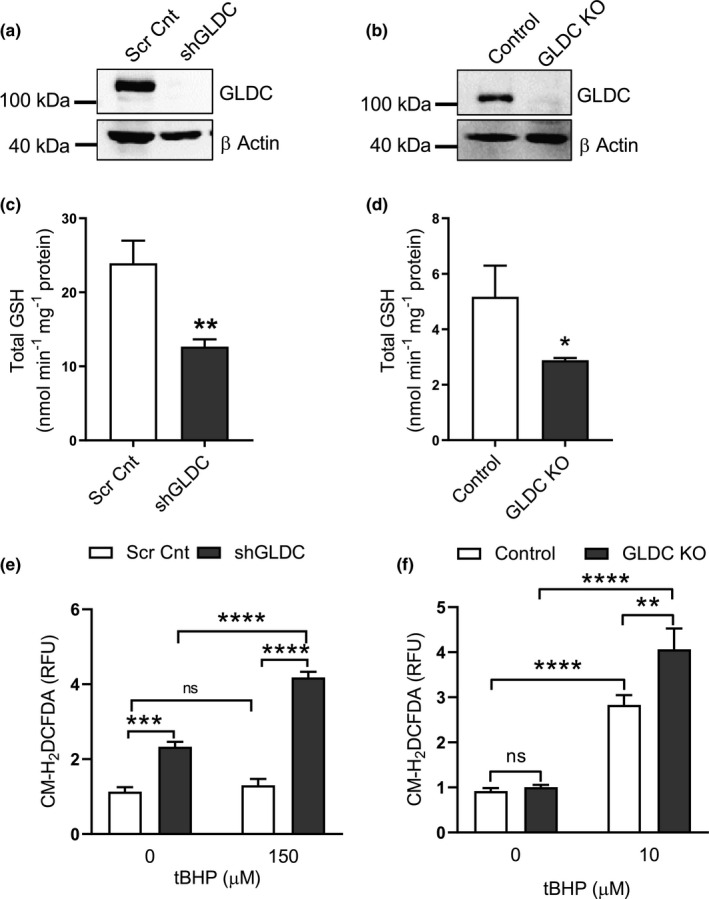
Suppression of GLDC expression reduces glutathione production and alters cellular redox balance. (A & B) Western blots showing the knockdown of GLDC in HepG2 cells stably transfected with control (scr cnt) or GLDC (shGLDC) shRNAs (a) or HEK‐293A in which the GLDC gene has been knocked out (KO) using CRISPR/CAS9 technology. (C & D) Quantitative determination of total glutathione levels in HepG2 cells (c) or HEK293A cells (d). Glutathione levels were normalized to total protein. Data are means (n = 3) ± standard deviation; *p < 0.05, **p < 0.01 as calculated by the Student's t‐test. (E & F) Measurement of intracellular oxidative stress in HepG2 (e) and HEK293A (f) cell lines as indicated using CM‐H_2_DCFDA fluorescence normalized to total protein as described in Procedures. Some cells were treated with tert butyl hydroperoxide (tBHP) to increase oxidative stress as indicated. Data are means (n = 4) ± standard deviation; *p < 0.05, **p < 0.01, ***p < 0.001, ****p < 0.0001 as calculated by two‐way ANOVA.

Contrary to our expectations, knockdown of GLDC by shRNA in HepG2 cells, or by CRISPR/Cas9 mutagenesis in HEK293A cells, resulted in a significant reduction in total glutathione levels; 1.9‐fold (*p *= 0.004) and 1.8‐fold (*p *= 0.025), respectively (Figure 5 A‐D). These unexpected results suggested that, at least in these experimental conditions, cellular glycine levels are already in excess of what is needed for maximal glutathione synthase activity. A potential explanation for the positive correlation between GLDC activity and glutathione production is that one of the products of glycine degradation by GLDC indirectly contributes to glutathione production.

To determine if the GLDC‐mediated reduction of glutathione had an effect on cellular oxidative stress, we measured reactive oxygen species (ROS) levels in the HepG2 cells with the shRNA knockdown of GLDC and in HEK293A cells with the CRISPR/Cas9‐mediated GLDC knockout. Intracellular ROS levels were measured using the CM‐H_2_DCFDA (2’,7’‐dichlorofluorescin diacetate) dye which is a cell‐permeable probe for ROS levels. We also included a pro‐oxidant (tert‐butyl hydroperoxide) treatment to determine if GLDC knockdown reduced the cell's ability to mount a protective glutathione‐mediated response to oxidative stress.

As shown in Figure 5, ROS levels were 1.76‐fold (*p *= 0.0003) higher in the HepG2 cells with reduced GLDC levels, compared to controls (Figure 5E, left‐hand bars). In addition, these cells showed an increased sensitivity to an oxidative stress challenge compared to control cells that were able to maintain low levels of ROS in the presence of tert‐butyl hydroperoxide (Figure 5E). The results from the GLDC knockout in the HEK293A cell line were similar with an increased sensitivity to an oxidative stress challenge (Figure 5F). Together, these results indicate that the GLDC‐mediated changes in glutathione production reduced the cells’ ability to control oxidative stress and ROS levels.

**FIGURE 6:**
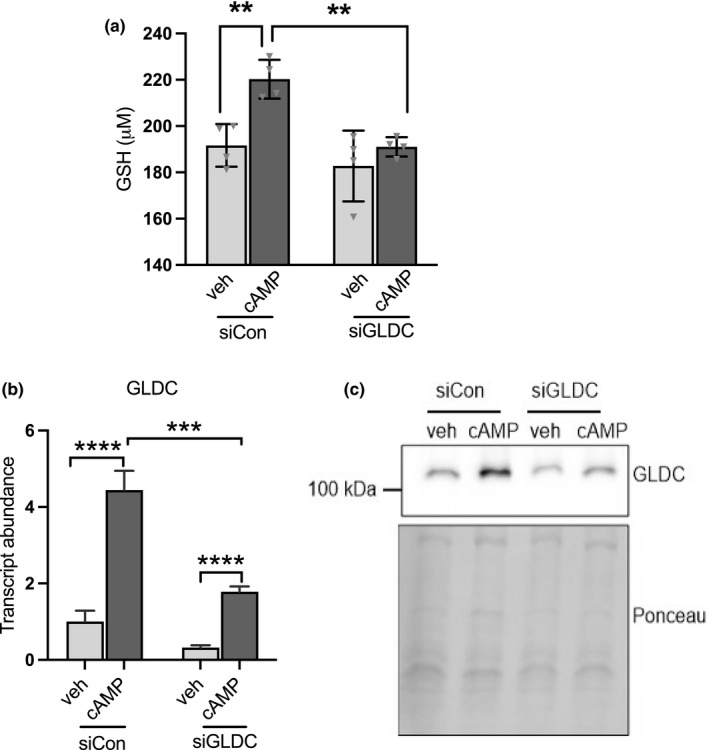
Stimulation of GLDC expression increases glutathione production. (a) Total glutathione levels in cAMP treated primary hepatocytes showing GLDC‐dependent stimulation of glutathione production. (b and c) GLDC transcript and protein levels in primary rat hepatocytes confirming siRNA‐mediated knockdown of cAMP‐stimulated GLDC gene expression. Data are means (n = 3) ± standard deviation; **p < 0.01, ***p < 0.001, ****p < 0.0001 as determined by two‐way ANOVA.

The above findings predict that elevated GLDC levels would cause an increase in glutathione levels. To test this possibility, we stimulated GLDC expression in primary rat hepatocytes using cAMP as described above (Figure 2) and measured total glutathione levels. As seen in Figure 6A and B, the stimulation of GLDC resulted in a significant increase in glutathione levels (Figure 6C). This cAMP‐mediated increase in glutathione was dependent on GLDC as shown by the siRNA‐mediated knockdown of GLDC, which abolished the stimulation of glutathione production (Figure 6C).

Taken together, our results suggest that elevated GLDC expression contributes to the reduced levels of circulating glycine seen in obesity and diabetes. Our findings also demonstrate that in addition to its effects on glycine levels, GLDC activity is functionally linked to glutathione production and the maintenance of cellular glutathione levels.

## 5 DISCUSSION

One of the major goals of this work was to determine if glycine decarboxylase (GLDC) has a metabolic function beyond the simple degradation of glycine. The scientific premise for asking this question was the longstanding observation that glycine levels are lower in diabetes and in pre‐diabetic states, which suggests a potential link between glycine metabolism and metabolic parameters associated with the development of diabetes. Given that GLDC is a key regulator of glycine levels in the body, we asked whether GLDC was in fact over‐expressed in diabetes and other metabolically stressed states, increasing glycine degradation and contributing to the observed reduction in circulating glycine levels seen in diabetes. Our results show that GLDC is, indeed, overexpressed in diabetic and obese rodents. These observations are consistent with a study that was published while this manuscript was in preparation showing that in ZDF rats, hepatic GLDC expression is also elevated by obesity (Simmons et al., 2020).

We then asked whether alteration of GLDC gene expression elicited physiological changes that might have an impact on the course of the disease. The findings reported here indicate that GLDC expression is strongly regulated by metabolic hormones and is indeed over‐expressed in animal models of diabetes and obesity. Additionally, our novel observation that GLDC expression is linked to glutathione production presents a possible mechanism for how altered GLDC expression might affect the progression of diabetes.

### 5.1 Regulation of GLDC by Glucagon

Our observation that GLDC gene expression is elevated in diabetic and obese states (Figure 1A‐C) raises the question as to what disease‐related signaling pathways mediate this regulation. Given that both glucagon and insulin were found to be strong regulators of GLDC expression (Figures 2 and 4), and that both hormones show altered levels in diabetes, the most likely explanation is that the disease‐related increase in GLDC expression is caused by elevated insulin or elevated glucagon, both of which can occur at different stages in the progression of diabetes. Another possible mechanism involving a specific microRNA may also contribute to a disease‐related elevation of GLDC expression. The miRNA‐30d‐5p has been shown to be a negative regulator of GLDC gene expression (Zhuang et al., 2019), and to be reduced in diabetes (Delic et al., 2016). It may be that a combination hormonal and miRNA signals lead to the upregulation of GLDC in metabolic diseases. In any case, these findings strongly indicate that the control of GLDC gene expression is integrated into the regulatory network that controls metabolic homeostasis and mediates physiological responses to metabolic stress and disease.

A curious aspect of our findings is that both glucagon and insulin stimulate the expression of GLDC. This is in contrast to many liver‐expressed genes that are counter‐regulated by the two hormones, such as PEPCK and glucose‐6‐phosphatase, both of which are stimulated by glucagon and suppressed by insulin. One gene that appears to share a related regulatory profile to GLDC is FGF21, encoding a hepatokine that regulates carbohydrate and lipid metabolism in response to fasting (Samms et al., 2017). FGF21 is cooperatively stimulated by both glucagon and insulin (Alonge et al., 2017). Although GLDC is similar in that it is positively regulated by both hormones, we did not observe a cooperative effect of the two hormones. For GLDC, the regulation by glucagon is much stronger than insulin, and this appears to be the dominant hormonal regulator of GLDC. This is consistent with the histological pattern of GLDC expression in the liver, where it is expressed primarily in periportal hepatocytes (Braeuning et al., 2006), which are the hepatic cells most responsive to glucagon. Even if glucagon is the primary metabolic signal stimulating GLDC expression, there are presumably physiological circumstances where insulin plays a role in GLDC expression. One possibility is that insulin functions to ensure a basal level of expression of GLDC when glucagon is not present, for example, after a meal.

Our conclusion that glucagon is a major regulator of GLDC expression was derived entirely from *in vitro* cell culture studies. This conclusion is consistent with human studies showing that glucagon has a substantial impact on glycine metabolism. A study performed on 6 healthy normal weight human subjects demonstrated the effects of selective glucagon deficiency and excess on 21 plasma amino acids. One of the largest changes was observed in glycine levels which increased (+24%) with glucagon deficiency and decreased (−20%) with glucagon excess (Boden et al., 1984).

The observation that GLDC is strongly regulated by metabolic hormones, as well as nutritional status, raises the possibility of a functional role for GLDC and glycine degradation that extends beyond the simple degradation of glycine. Our finding that GLDC expression is linked to glutathione production is consistent with this idea.

### 5.2 Physiological effects of altered GLDC expression

Given the well‐known requirement for glycine in the synthesis of glutathione, we originally formulated a hypothesis in which reduced availability of glycine incurred by elevated GLDC and increased glycine degradation would limit glutathione biosynthesis. Based on this, we expected that when GLDC expression was reduced, glycine availability would increase (due to reduced glycine degradation), and that this would stimulate glutathione production. Given these expectations, we were surprised to observe the opposite effects. Reduction of GLDC caused the suppression of total glutathione levels (Figure 5A‐D), while overexpression of GLDC was associated with an increase in total glutathione (Figure 6).

Notably, these findings were derived from in vitro systems where glycine is likely to be in excess with regard to glutathione synthase, the enzyme that adds glycine to the cysteine–glutamate dipeptide to form glutathione. Under these conditions, the reduced glutathione production caused by the loss of GLDC must be caused by some other components of glycine catabolism. One possibility is that a product of glycine degradation reaction plays a role in glutathione biosynthesis. When glycine is degraded by GLDC, one‐carbon units in the form of methylenetetrahydrofolate are produced and enter the mitochondrial folate one‐carbon cycle. The mitochondrial one‐carbon cycle could be indirectly linked to glutathione production via the transfer of folate to the cytosol where it ultimately engages the transsulfuration pathway that is linked to cysteine production, potentially influencing the first step in glutathione synthesis (Beatty & Reed, 1980; Gregory et al., 2000; Kaplowitz et al., 1985; Pike et al., 2010; Santos et al., 2020). Metabolic tracing experiments will be required to directly test this possibility.

These findings are similar to previous observations from hepatoma cells where GLDC knockdown caused a decrease in GSH/GSSG ratio and an increase in cellular ROS (Zhuang et al., 2018). However, they are in contrast to a previous study showing a decline in cellular GSH levels with db‐cAMP treatment in rat hepatocytes (Lu et al., 1991). Although we do not fully understand the basis for this discrepancy there were substantial differences in the growth medium, the time of treatment, and GSH measurement methods between this study and ours.

Another interesting question raised by our findings is why glucagon stimulates GLDC expression so strongly. What is the physiological consequence or function of this stimulation?

Presumably, the answer to this question is closely related to the principal metabolic outcomes of elevated GLDC activity: reduced glycine levels, increased production of the byproducts of glycine cleavage (1‐carbon groups, and NADH), and increased glutathione production.

Glucagon is typically elevated during a fast. The fasted state is also associated with increased hepatic mitochondrial energy production (Halestrap, 1982; Yamazaki, 1975; Yamazaki et al., 1980). It may be that under these conditions the glucagon‐mediated stimulation of GLDC contributes to mitochondrial energy output by increasing the availability of NADH (a product of GLDC action), which could serve as an electron donor to oxidative phosphorylation. NADH could also be transferred to the cytosol (via the malate‐oxaloacetate shuttle) where it could contribute to the cytosolic NADH pool required for gluconeogenesis (Siess et al., 1977).

Another possible role for the glucagon‐mediated stimulation of GLDC could be related to the special circumstances that occur after a pure protein meal, where in addition to insulin, glucagon levels are elevated (Delic et al., 2016). The resulting glucagon‐mediated stimulation of GLDC under these conditions could be related to the need to manage the postprandial elevation of circulating amino acids by increasing the degradation and disposal of glycine.

### 5.3 Role of GLDC expression in disease

The observation that formed the impetus for this study was the reduced plasma glycine seen in diabetic individuals. Although our findings indicate that the stimulation of GLDC activity in diabetes and obesity is likely to contribute to the reduced glycine levels seen in these states, the possibility that changes in GLDC expression influence the susceptibility to or progression of diabetes remains an open question. One speculative possibility is that the elevation of GLDC in diabetic and pre‐diabetic states is a type of compensatory activity that increases glutathione production as a protective role in the pro‐oxidative environment of diabetes.

In addition to its apparent link to diabetes, GLDC gene expression has been shown to be elevated in many cancers, including non‐small cell lung cancer (Zhang et al., 2012), breast cancer (Kwon et al., 2014), glioblastoma multiforme (Kim et al., 2015), and prostate adenocarcinoma (Woo et al., 2018). How these observations integrate with the proposed role of GLDC in metabolic disease is a question that remains to be answered.

In summary, our findings indicate that the expression of the glycine degrading enzyme GLDC is elevated by metabolic stress related to diabetes and suggest that the reduced levels of glycine seen in diabetes and obesity are due at least in part to elevated GLDC expression. Given the key role of GLDC in determining glycine levels and the potential role of glycine in metabolic health, our novel finding that GLDC gene expression is strongly regulated by metabolic hormones is consistent with the idea that GLDC plays a role in general metabolic regulation. Finally, the linkage between GLDC expression, glutathione production, and cellular oxidative stress suggests a potential mechanism by which altered glycine metabolism could influence the development of diabetes.

## Supporting information



Fig S1
