# Supplementary material for: Hormonal regulation of glycine decarboxylase and its relationship to oxidative stress
Source: Physiol Rep. 2021 Aug 2;9(15):e14991. doi: 10.14814/phy2.14991 (PMC8329434; doi:10.14814/phy2.14991)
Supplement: Supplementary file 1 — Fig S1 [file PHY2-9-e14991-s001.docx]

Primers used in the RT-qPCR analysis

| **Primer Name** | **Sequence** |
| --- | --- |
| Mouse GLDC forward | CATGATTGAGCCCACCGAGT |
| Mouse GLDC reverse | TGGAGACATCTTCAGGGGGT |
| Mouse FASN forward | GACTCGGCTACTGACACGAC |
| Mouse FASN reverse | CGAGTTGAGCTGGGTTAGGG |
| Mouse PCK1 forward | GTTTGATGCCCAAGGCAACT |
| Mouse PCK1 reverse | CCTTCCCAGTAAACACCCCC |
| Mouse PPIA forward | GCGTCTCCTTCGAGCTGTT |
| Mouse PPIA reverse | CTCTCCGTAGATGGACCTGC |
| Rat GLDC forward | GGAGGGGTCTTCTAGGGTCT |
| Rat GLDC reverse | CAGGTGATGGGCAGTGGAAT |
| Rat FASN forward | TCGACTTCAAAGGACCCAGC |
| Rat FASN reverse | ACTGCACAGAGGTGTTAGGC |
| Rat PCK1 forward | CCCAAGAGCAGAGAGACACC |
| Rat PCK1 reverse | CATACATGGTGCGGCCTTTC |
| Rat TBP forward | CCCCGGTGGAAGACAGTTTTA |
| Rat TBP reverse | CCAAGCCCTGAGCATAAGGT |
| Human PPIA forward | GTCTCCTTTGAGGTAAGGGGC |
| Human PPIA reverse | GCTGCACGATCAGGGGTAA |
| Human GLDC forward | GGCCCATCGGAGTGAAGAAA |
| Human GLDC reverse | TATCGCAGTTTCCGTGGCTT |
| Rat PPIB forward | TCCGTGGCCAACGATAAGAAG |
| Rat PPIB reverse | GCCAAATCCTTTCTCTCCTGTAGC |
| Rat CREB1 isoform A forward | TGAAGAACAGGGAAGCAGCAAGAG |
| Rat CREB1 isoform A reverse | TTGGTTTTTAAGCACTGCCACTCTG |
| Rat CREB1 isoform B forward | ACTGAGGAGCTTGTACCACCG |
| Rat CREB1 isoform B reverse | CTGGCATGGATACCTGGGCT |
| Rat ATF1 forward | ATCCCAGCATTTCTGCCGTC |
| Rat ATF1 reverse | ATGGCAATGTACTGTCCGCTG |
